# Antioxidant Activity and Resistance Against Oxidation of Peptide Fractions from Common Bean (*Phaseolus vulgaris* L.) Landraces Assessed by EPR and Chemical Assays

**DOI:** 10.3390/antiox15030376

**Published:** 2026-03-18

**Authors:** Katherine Márquez-Calvo, Guillermo Schmeda-Hirschmann, Felipe Leyton, Felipe Ávila, Pablo Salgado, Victoria Melin, David Contreras, Gipsy Tabilo-Munizaga

**Affiliations:** 1 Department of Food Engineering, Faculty of Health and Food Science, University of Bío-Bío, Av. Andrés Bello 720, Chillán 4051381, Chile; gtabilo@ubiobio.cl; 2 Laboratorio de Química de Productos Naturales, Instituto de Química de Recursos Naturales, Universidad de Talca, Campus Lircay, Talca 3480094, Chile; schmeda@utalca.cl (G.S.-H.); frleyton@uc.cl (F.L.); 3 Department of Nutrition and Food Science, School of Nutrition and Dietetics, Health Science Faculty, Campus Lircay, University of Talca, Talca 3480094, Chile; favilac@utalca.cl; 4 Departamento de Ingeniería Civil, Facultad de Ingeniería, Universidad Católica de la Santísima Concepción, Concepción 4090541, Chile; psalgado@ucsc.cl; 5 Departamento de Química Analítica e Inorgánica, Facultad de Ciencias Químicas, Universidad de Concepción, Campus Universitario, Concepción 4070409, Chile; victoriamelin@udec.cl (V.M.); dcontrer@udec.cl (D.C.)

**Keywords:** food proteins, bioactive peptides, antioxidant peptides, legume valorization, plant protein ingredients, *Phaseolus vulgaris*

## Abstract

The objective of this study was to obtain and characterize bioactive peptides derived from common bean (*Phaseolus vulgaris*) landraces and to evaluate their antioxidant potential using multiple in vitro assays. Protein isolates were obtained by isoelectric precipitation followed by enzymatic hydrolysis using Alcalase. Peptides were separated by ultrafiltration into fractions < 3 kDa and 3–10 kDa, yielding a total of forty samples. Antioxidant activity was evaluated using DPPH, FRAP, and ORAC assays. Antioxidant responses ranged from 13.06 to 50.8% inhibition in DPPH, 52.2 to 1750 µmol TE/100 g in FRAP, and 305 to 5246 µmol TE/100 g in ORAC. Resistance against oxidation ranged from 10.6 to 68.8%. Peptides < 3 kDa generally exhibited higher antioxidant activity in the functional assays, particularly in the Apolo, Magnum, Boloto, and Hallado landraces, although some 3–10 kDa fractions also showed relevant activity. Peptide extraction yields ranged from 3.73 to 10.39% and from 1.33 to 4.74%, while soluble protein contents ranged from 23.1 to 460 and from 9.9 to 288 mg BSA/100 g beans for <3 kDa and 3–10 kDa fractions, respectively. Overall, the results support the potential of common bean-derived peptides as functional food ingredients with antioxidant activity mediated through multiple mechanisms.

## 1. Introduction

Diet has been widely associated with Non-Communicable Diseases (NCDs), as poor nutrition is a risk factor for these types of diseases [1,2]. One key mechanism involved in the development of NCDs includes Reactive Oxygen Species (ROS), which are metabolic factors [3]. ROS act as signaling molecules or cause oxidative damage in tissues, depending on the balance between ROS production and its elimination. ROS and oxidative stress have been related to the development of NCDs [4]. ROS come from endogenous and exogenous sources, with diet being a modifiable exogenous source [5]. Therefore, a higher consumption of fruits, vegetables, and legumes is associated with a lower incidence of NCDs [6]. Also, the world population has been increasing the demand for protein from various plant sources, with pulses being particularly noteworthy, as these provide multiple benefits for human health and the environment [7,8].

The genus *Phaseolus*, in particular, includes five domesticated species, with *Phaseolus vulgaris* (common bean) standing out as the most important grain for direct human consumption [9,10]. The domestication of the common bean resulted in six races originating from two major genetic pools: the Mesoamerican pool, which includes the Durango, Jalisco, and Mesoamerica races, and the Andean pool, comprising the Nueva Granada, Perú, and Chile races. Among these, the Chilean race is considered the least characterized, despite exhibiting a high degree of phenotypic diversity [11]. This diversity may be partly explained by the wide environmental gradient across Chile, where common bean landraces are cultivated from extremely arid northern regions to humid southern zones, conditions that have promoted local adaptation and the distinctive phenotypic traits of the Chilean race [11].

Previous studies, including recent contributions from our group, have advanced the characterization of Chilean landraces, particularly in terms of their phenolic compounds, amino acids, and lipid profiles [12,13,14,15,16]. However, the characterization of protein and peptide fractions in this race remains largely unexplored.

Common beans are an important source of protein, providing essential amino acids, complex carbohydrates, and dietary fiber [17,18]. In this context, in vitro, in vivo, and clinical evidence have shown that regular consumption of common beans is associated with a reduced risk of NCDs, including diabetes and cancer, and presents with antioxidant activities [19,20,21]. These bioactive properties have been associated with compounds such as polyphenols, saponins, and carotenoids [13,16].

Protein and peptide also contribute to these bioactive properties [17,22]. The main storage protein in common beans is phaseolin, which has limited nutritional value due to its low methionine and cysteine content and high resistance to proteolysis [9,23]. While many peptides remain inactive within the protein sequence, specific bioactive peptides can be released through enzymatic hydrolysis, either in vivo during digestion or in vitro under controlled conditions, enhancing physiological functions [24,25,26]. For example, peptide fractions and protein hydrolysates from common beans have been shown to reduce inflammation, oxidative stress, and cardiovascular risk factors in animal models [25,26]. Recently, various investigations have focused on the bioactive properties of peptides obtained from legumes [22,27,28]. In this way, enzymatic processes enhance the biological potential of bean protein by producing protein isolates and bioactive peptides [17,22,28,29,30]. These peptides exhibit antioxidant activity and can inhibit lipid accumulation, obesity, inflammation, and hypertension. These also offer advantages over small-molecule drugs, including broad action, structural diversity, low toxicity, and tissue accumulation [31]. In this context, this research aims to characterize peptide fractions obtained from common bean landraces and evaluate their antioxidant potential using complementary analytical approaches, such as DPPH, ORAC, and FRAP assays, along with resistance against oxidation assessed by electron paramagnetic resonance. This study investigates the hypothesis that peptides from Chilean common bean landraces could act as natural inhibitors of oxidative processes and that their bioactivity is highly dependent on their genotype. Overall, this work aims to contribute to a better understanding of the functional properties of common bean peptides and to select the best bean landraces for their subsequent enhancement in the development of functional ingredients.

## 2. Materials and Methods

### 2.1. Samples

**Figure 1:**
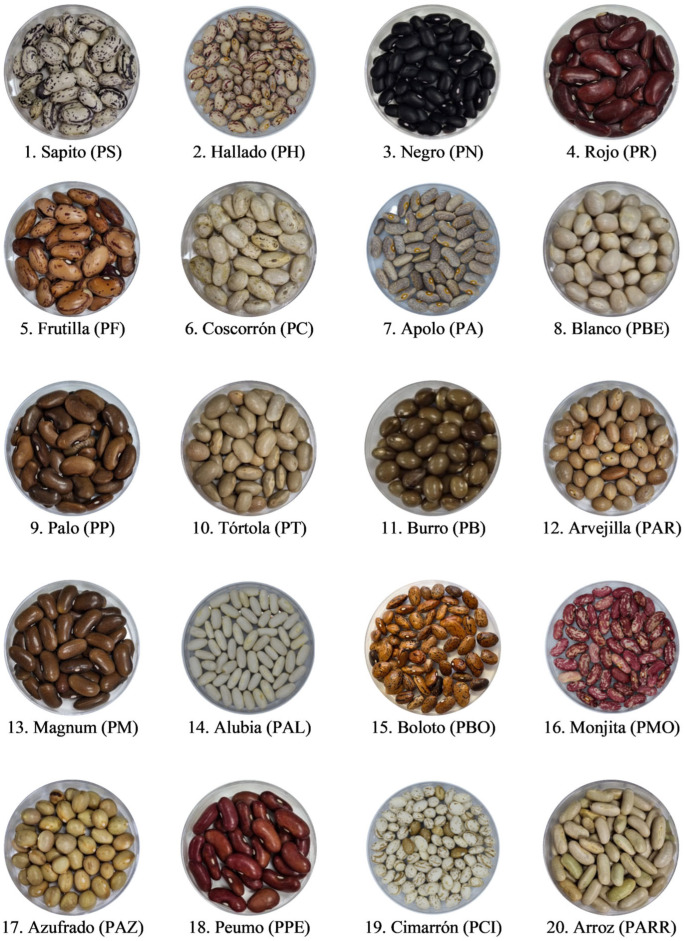
The twenty Chilean common bean (*Phaseolus vulgaris*) landraces used for the present study, with their full landrace name and its corresponding acronym (in parentheses).

The twenty known landrace common beans were collected from the Ñuble to the O´Higgins Region, Chile in 2023, and whole beans are shown in Figure 1. Landrace common beans were transported to the laboratory and stored at 4 °C until processing. Moisture content was measured in triplicate using a thermobalance (moisture analyzer) at 105 °C. This approach follows the principle of moisture determination in seeds by recording weight loss during controlled heating, as described in the seed moisture literature [32]. For sample processing, whole beans were pulverized (425 µm) using a POWTEQ FM200 ultracentrifugal mill (Beijing, China) to obtain ground bean samples.

### 2.2. Standards and Reagents

Gallic acid, vanadate-molybdate reagent, azomethine-H, 1,10-phenanthroline, acetic acid, potassium phosphate monobasic, potassium phosphate dibasic, hydroxybenzene, Folin–Ciocalteu’s phenol reagent, 2,2′-Azobis(2-methylpropionamidine) dihydrochloride, sodium azide, sodium fluorescein, 1,1-Diphenyl-2-picrylhydrazyl, ethanol, methanol, reagents and solvents were supplied by Sigma Aldrich, Steinheim, Germany. Ethanol, o-phosphoric acid, sodium hydroxide, sulfuric acid 96%, sodium carbonate, acetone, 6-Hydroxy-2,5,7,8-tetramethylchroman-2-carboxylic acid (Trolox), and centrifugal filters Amicon Ultra-15 (10,000 NMWL; 3000 NMWL) were supplied by Merck Millipore, Burlington, MA, USA. The purity of all standards was higher than 95%, and ultra-pure water was obtained from a Milli-Q system (Millipore, Milford, MA, USA).

### 2.3. Protein Extraction

Proteins were extracted based on the literature, adapted to common beans [27]. Briefly, ground bean samples were suspended in distilled water (1:9, *w*/*v*) adjusted to pH 9.0 with NaOH 1 mol/L and agitated for 120 min at 25 °C on a multiple shaker (Scilogex MS-M-S10, Rocky Hill, CT, USA). Then the samples were centrifuged at 4500 rpm (≈2280× *g*) for 10 min at 20 °C using a refrigerated centrifuge (Bioprocen 22 R, Ortoalresa, Madrid, Spain; rotor radius = 10.1 cm).

**Figure 2:**
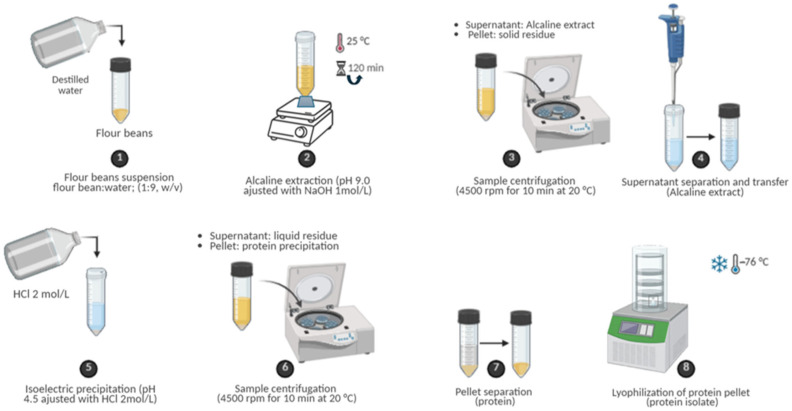
Schematic representation of alkaline extraction and isoelectric precipitation workflow for protein isolate recovery from common bean flour.

After centrifugation, the supernatant corresponding to the alkaline extract (soluble protein fraction) was carefully collected, whereas the pellet (insoluble residue) was separated and stored for potential future studies. The recovered supernatant was adjusted to pH 4.2 using 2 mol/L HCl to induce isoelectric precipitation of proteins. The samples were centrifuged again under the same conditions to separate the precipitated proteins (pellet) from the remaining liquid fraction. Finally, the protein pellet was collected and lyophilized using a freeze-dryer (Biobase BK-FD10PT, Wolfenbuettel, Germany) to obtain protein isolates. Each bean sample was processed in triplicate (see Figure 2).

### 2.4. Peptide Fractions

The lyophilized protein isolates were dissolved in distilled water at an initial solid-to-liquid ratio of 10 g/100 mL and digested with Alcalase using an enzyme-to-substrate ratio of 0.3 AU/g at pH 8 and 50 °C for 60 min. Hydrolysis was carried out in a 1000 mL beaker equipped with magnetic stirring, temperature, and pH control. Alcalase was inactivated by heating the reaction mixture at 85 °C for 15 min [33]. Bean hydrolysates were separated by centrifugation for 30 min at 4500 rpm (≈2280× *g*) at 25 °C, and the supernatant was collected.

**Figure 3:**
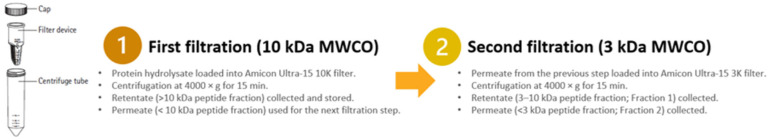
Schematic representation of the sequential ultrafiltration procedure used to obtain peptide fractions from bean protein hydrolysates using Amicon Ultra-15 centrifugal filters.

The supernatant was subsequently fractionated by sequential ultrafiltration using Amicon Ultra-15 centrifugal filter devices (Millipore) with nominal molecular weight cut-offs (MWCO) of 10 kDa and 3 kDa. The supernatant was first loaded onto the 10 kDa device and centrifuged at 4000× *g* for 15 min. The permeate (<10 kDa) was collected and further filtered through the 3 kDa device under the same conditions. The retentate of the 3 kDa device corresponded to the 3–10 kDa Fraction 1, while the permeate corresponded to the <3 kDa Fraction 2 (see Figure 3). These fractions were selected for subsequent bioactivity analyses based on previous reports indicating that low-molecular-weight peptides frequently exhibit enhanced antioxidant activity [28,33]. The peptides were lyophilized. Before and after digestion with Alcalase, the protein was evaluated by Dumas method.

### 2.5. Protein Content by Dumas and Bradford Methods

Total nitrogen was determined according to Dumas method using a factor of 6.25 for the determined total protein content in legumes according to the literature [16]. Although legume-specific factors have been proposed, this conventional value was selected to ensure comparability with previously published studies on protein isolates and hydrolysates from legumes and other functional protein sources.

Soluble protein was quantified using the Bradford method using the Bio-Rad Protein Assay (Bio-Rad Kit I 5000001, Hercules, CA, USA), following procedure from the literature [34]. Peptide fractions and standards were prepared in triplicate. A calibration curve was generated using BSA (8–80 µg/mL, r^2^ = 0.998) and a blank, consisting of all reagents except sample or standard, and was included to correct for background absorbance. All determinations were carried out in triplicate, and the results are reported as the arithmetic means ± SD.

### 2.6. SDS-PAGE and Densitometry Analysis

Protein isolates were separated by SDS-PAGE (Sodium Dodecyl Sulfate Polyacrilamide Gel Electrophoresis) using a discontinuous buffer system originally described by Ulrich K. Laemmli [35]. Resolving gels (15% acrylamide, pH 8.8) and stacking gels (4% acrylamide, pH 6.8) were prepared according to standard protocols. Electrophoretic separations were performed using a Bio-Rad Mini-Protean II electrophoresis system (Bio-Rad, USA) with a constant voltage program of 90 V per gel for 15 min followed by 150 V for 90 min. Samples (15 μL per well) were loaded together with a molecular weight marker covering the 4.6–25 kDa range (SeeBlue Plus2 Pre-stained Protein Standard, Thermo Fisher Scientific, Waltham, MA, USA), which was used to define the molecular mass ranges and verify band positions.

Gels were stained with 0.1% (*w*/*v*) Coomassie Brilliant Blue R-250 in 40% (*v*/*v*) methanol and 7% (*v*/*v*) acetic acid for 40 min and destained in 40% methanol and 10% acetic acid until clear background was achieved. For comparative interpretation of electrophoretic profiles, reference lanes containing bovine serum albumin (BSA) and the enzyme preparation (Alcalase) were loaded alongside peptide samples. Band intensities were quantified by densitometry using ImageJ software (v. 1.54p, NIH, Bethesda, MD, USA). Each lane was quantified considering band intensities within molecular mass ranges of 4.6–10, 10–15, 15–25, and >25 kDa, and normalized to the total band intensity within the same lane.

### 2.7. Antioxidant Activity Determinations

The forty peptide fractions obtained from the <3 kDa and 3–10 kDa ultrafiltration of the protein hydrolysates were evaluated for antioxidant activity by three methods, following established protocols (see below).

#### 2.7.1. ORAC Assay

A microplate reader (Tecan Infinite M Nano+, Mannedorf, Switzerland) was used to analyze antioxidant capacity by ORAC assay. Peptide fractions were evaluated at an initial concentration of 0.05 mg/mL, and black 96-well plates were employed for the measurements. Briefly, 150 μL of 0.11 μM fluorescein was added to each well, followed by 25 µL of the peptide fractions. All solutions and samples were dissolved in phosphate buffer (75 mM, pH 7.4). The 2,2′-azobis(2-methylpropionamidine) dihydrochloride (AAPH, 152 mM) radical was activated for 30 min at 37 °C in the plates. Finally, 25 μL of activated AAPH was added to each well containing the fluorescein and samples or standard or blank. Fluorescence decay was recorded for 90 min. Differences in the areas under the fluorescence decay curve (AUC) between the blank and the sample were calculated over time, and the results were expressed as μmol Trolox equivalents per 100 g of beans [36]. Quantification was performed using a Trolox standard curve (0–50 μM; r^2^ = 0.998) and the blank consisted of phosphate buffer without any sample. All determinations were performed in triplicate, and results are reported as arithmetic means ± SD.

#### 2.7.2. DPPH Radical Scavenging Activity Assay

The free radical scavenging activity of peptide fractions was determined using the DPPH assay, based on slightly modified protocol from the literature [37]. Peptide fractions were dissolved in methanol at 1 mg/mL and kept in the dark. Briefly, 20 μL of the samples were mixed with 180 μL of DPPH 100 µM (prepared daily in pure methanol) in a microplate. A blank, consisting of DPPH solution without any sample, was included to correct for background absorbance. A control was also prepared with 100 μL methanol + 180 μL DPPH, representing the maximum reference absorbance. The reaction mixture was incubated for 30 min at 37 °C, and absorbance was measured at 517 nm using a microplate reader (Spectrostar Nano, BMG Labtech, Ortenberg, Germany). A calibration curve of Trolox (5–100 µmol/L, r^2^ = 0.971) was used to determine the Trolox equivalents in the samples. Results are expressed as % Inhibition at 380 µg/mL. All determinations were carried out in triplicate, and the results are reported as arithmetic means ± SD.

#### 2.7.3. Ferric Reducing Antioxidant Power (FRAP) Assay

The FRAP assay was carried out as reported by Benzie and Strain 1996 [38]. Peptide fractions were dissolved in 80% methanol (*v*/*v*) to a final concentration of 700 µg/mL. Then, 200 µL of the samples were mixed with 1.5 mL of FRAP reagent and incubated at 37°C for 30 min. Absorbance was measured at 595 nm using a microplate reader (Spectrostar Nano, BMG Labtech, Ortenberg, Germany) with a 10 mm plastic cuvette. A blank, consisting of FRAP reagent without any sample, was included to correct for background absorbance. A calibration curve of Trolox (25–250 µmol/L, r^2^= 0.999) was used to determine the Trolox equivalents (TE) in the samples. Results are expressed as µmol TE per 100 g of beans. All determinations were carried out in triplicate, and results are reported as arithmetic means ± SD.

### 2.8. Measurement of Peptide Resistance Against Oxidation Using EPR

The reduction in radical products from the Fenton reaction using 5,5-dimethyl-1-pyrroline-N-oxido (DMPO) as a spin trap was determined by Electron Paramagnetic Resonance (EPR) assays, adapted from the literature [39,40]. The antioxidant activity of forty peptide fractions from bean landraces was evaluated. The spectra were measured in a Bruker model EMX micro-spectrometer (Bruker GmbH, Mannheim, Germany). Hydroxyl radical (^●^OH) was generated by the Fenton reaction [39], with the reaction mixtures containing 200 μL of 10 mM H_2_O_2_, 200 μL of 10 mM FeSO_4_, and 200 μL of 300 mM DMPO in 10 mM PBS Buffer at pH 7.4. Aliquots of 20 μL of 1.0 mg/mL peptides were mixed with 60 μL of the above reaction solution and then transferred into 100 μL capillary micropipettes (Blaubrand, Intramark, Brand GmbH, Wertheim, Germany), and an EPR spectrum was recorded 2.5 min later. The instrumentation and conditions were as follows: EPR microwave power: 2.000 mW, attenuation: 20 dB, sweep time: 30 s, number of scans: 10; receiver gain: 30 dB; sweep width: 60 G, and 3505 G as the center field for experiments with DMPO as spin trapping was used.

A Fenton control, consisting of the reaction mixture without any peptide sample, was included as the reference for maximum radical formation and to determine the % inhibition of ^●^OH by the peptides. Signal quantification was performed using the double integral under the peak, and the results were expressed as the signal intensity and the area values. All determinations were carried out in triplicate, and the results are reported as the means ± SD.

### 2.9. Statistical Analysis

Statistically significant differences in compound content and antioxidant capacity were evaluated using one-way ANOVA followed by Dunnett’s test (*p* < 0.01) in GraphPad Software Prism 8 (San Diego, CA, USA). Prior to analysis, assumptions of normality and homogeneity of variances were assessed. When both assumptions were met, a standard one-way ANOVA followed by Dunnett’s post hoc test was applied. When variance homogeneity was violated, Kruskal–Wallis test followed by Dunn’s multiple comparisons test was used. For each assay (DPPH, ORAC, FRAP, and EPR), comparisons were performed against a specific reference sample selected within each dataset as the control, defined as the variety exhibiting the highest bioactivity for the corresponding assay. Datasets meeting normality assumptions were additionally analyzed using Pearson’s correlation test to evaluate linear relationships between soluble protein content and antioxidant activity.

## 3. Results

### 3.1. Composition of Common Bean Flours and Protein Isolates

The initial protein content, expressed on a dry weight (DW) basis, ranged from 15.18 to 22.75 g/100 g of beans for PT and PCI landraces, respectively. Protein isolates obtained from these twenty landraces had concentrations between 59.8 and 81.3 g/100 g DW for PP and PARR landraces. Bean extraction yields ranged from 15.32% and 26.40% for PT and PCI landraces, while protein yield ranged from 55.75% and 77.9% for PS and PMO landraces (see Table 1).

### 3.2. Densitometry Analysis of Protein Isolates

**Figure 4:**
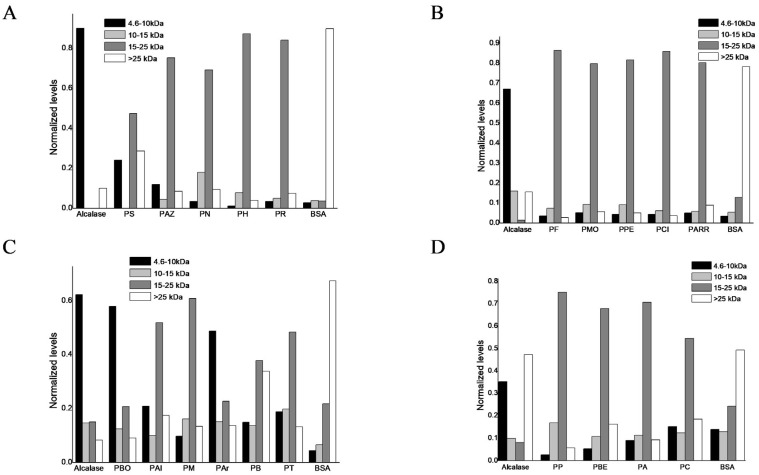
Densitometric analysis of protein isolated from common bean landraces digested with Alcalase and filtered out with ultrafiltration with a cut-off of 3–10 kDa. Bars represent the distribution of the protein according to its molecular mass. The x-axis corresponds to individual lanes (peptide samples, BSA, and Alcalase as reference lanes), and the y-axis represents relative band intensity normalized to the total signal within each lane. Subfigures correspond to different sets of landraces: (**A**) PS, PAZ, PN, PH, and PR; (**B**) PF, PMO, PPE, PCI, and PARR; (**C**) PBO, PAL, PM, PAR, PB, and PT; (**D**) PP, PBE, PA and PC.

The protein hydrolysates from the twenty common bean landraces, obtained after alkaline solubilization and enzymatic hydrolysis with Alcalase, were quantitatively analyzed by densitometry. BSA was included as a control as a molecular mass reference protein. The relative abundance of proteolyzed fractions was determined to characterize the molecular mass distribution of polypeptides from protein hydrolysates within the ranges of 4.6–10, 10–15, 15–25, and >25 kDa. In Figure 4, the y-axis represents the relative band intensity (%) corresponding to each molecular mass range, calculated by densitometric normalization to the total band intensity per lane.

Figure 4A shows densitometry for PS, PAZ, PN, PH, and PR landraces. Figure 4B shows the densitometry analysis for PF, PMO, PPE, PCI, and PARR. Figure 4C shows the densitometry analysis for PBO, PAL, PM, PAR, PB, and PT. Figure 4D. shows the densitometry analysis form for PP, PBE, PA, and PC. Figure 4D shows that 15–25 kDa are the most abundant polypeptides in PS, PAZ, PN, PH, PR, PF, PMO, PPE, PCI, PARR, PAL, PM, PB, PT, PP, PBE, PA, and PC landraces. Although the 15–25 kDa fraction represents the most abundant polypeptides, soluble protein content and bioactivity analyses were performed only for the <3 kDa and 3–10 kDa fractions, as low-molecular-weight peptides are generally associated with higher biological activity [22,41,42]. The major storage protein phaseolin (47–49 kDa) [43] was absent in all protein hydrolyzed fractions, indicating extensive hydrolysis by Alcalase and supporting the expected cleavage of globulins into polypeptides between 15 and 25 kDa according to the literature [44]. For the PBO and PAR samples, the most abundant polypeptides were distributed within the 4.6–10 kDa molecular mass range, accounting for approximately 58% and 49% of the total bands detected by SDS-PAGE, respectively (Figure 4C). Peptides <3 kDa were not visualized, which is consistent with the intrinsic resolution limit of conventional Laemmli (glycine) SDS-PAGE systems for very-low-molecular-weight peptides. Electrophoresis was not intended to detect or validate peptides below 3 kDa, but rather to visualize peptide distribution within the resolvable molecular weight range.

### 3.3. Composition of Peptide Fractions

#### Soluble Protein Content of Peptide Fractions

**Figure 5:**
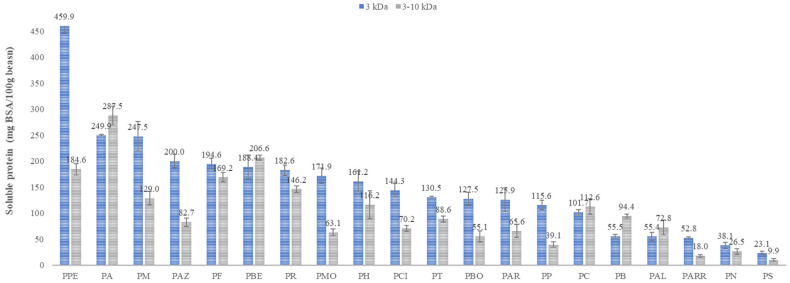
Soluble protein content in forty peptide fractions.

The soluble protein content quantified through the Bradford method showed clear variability among landraces and between the two molecular masses. For the 3–10 kDa peptide fractions, soluble protein ranged between 9.9 and 287.5 mg BSA/100 g beans DW, for PS and PA landraces, respectively (Figure 5). For the 3 kDa peptide fractions, soluble protein ranged between 23.1 and 459.9 mg BSA/100 g beans DW for PS and PPE landraces, respectively (Figure 5).

### 3.4. Bioactivities of Peptide Fractions Mediated by Multiple Mechanisms

#### 3.4.1. DPPH, FRAP and ORAC Assays

The antioxidant activities of peptide fractions across landraces and molecular weight ranges are summarized in Table 2. DPPH inhibition ranged from 12.87 to 50.8% for the 3–10 kDa fractions and from 12.87 to 39.6% for the <3 kDa fractions. FRAP values varied from 52.2 to 712 µmol TE/100 g beans in the 3–10 kDa fractions and from 180.7 to 1750 µmol TE/100 g beans in the <3 kDa fractions. ORAC values ranged from 305 to 1838 µmol TE/100 g beans for the 3–10 kDa fractions and from 868 to 5246 µmol TE/100 g beans for the <3 kDa fractions. Statistical analyses indicated significant differences among landraces for all antioxidant assays (Table S2, Supplementary Materials). Overall, the <3 kDa fractions generally exhibited higher antioxidant capacities than the 3–10 kDa fractions, with minor exceptions in DPPH and FRAP assays. Peptide extraction yields, calculated relative to the initial dry seed mass, ranged from 3.73 to 10.39% for the <3 kDa fractions and from 1.33 to 4.74% for the 3–10 kDa fractions, indicating higher recovery efficiency for lower molecular weight fractions.

#### 3.4.2. Resistance Against Oxidation Assessed by EPR

**Figure 6:**
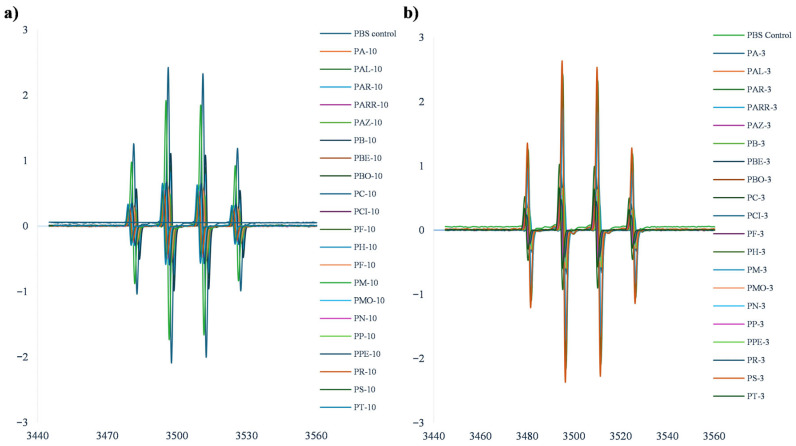
Resistance against oxidation determined by EPR. (**a**) EPR spectrum for 3–10 kDa peptide fractions; (**b**) EPR spectrum for 3 kDa peptide fractions.

The resistance against oxidation (RAO) of the forty peptide fractions obtained from the <3 kDa and 3–10 kDa ultrafiltration of the protein hydrolysates were evaluated by electron paramagnetic resonance (EPR) using DMPO for spin trapping. EPR spectra of 40 peptide fractions and their corresponding controls were recorded at a central magnetic field of 3510 G with a scan range of 100 G. In all samples, the characteristic DMPO–OH spin-adduct was detected (Figure 6a,b), confirming the generation of hydroxyl radicals under the experimental conditions. The hyperfine coupling constants obtained from the experimental spectra were consistent across all samples (aH = 14.70 and aN = 15.20), indicating the formation of the expected hydroxyl radical adduct. These values were in agreement with those reported for DMPO–OH adducts in aqueous systems.

Both peptide fractions (3–10 kDa and <3 kDa) produced a clear attenuation of the DMPO–OH signal compared to the phosphate-buffered saline (PBS) control, indicating their capacity to reduce hydroxyl radical availability. The extent of signal attenuation varied among landraces and molecular weight fractions. In general, the <3 kDa fractions exhibited a stronger reduction in the EPR signal than the corresponding 3–10 kDa fractions, indicating a higher resistance against oxidation.

Differences among landraces were also evident, with some samples showing a pronounced decrease in signal intensity, while others exhibited more moderate effects. These variations reflect differences in peptide composition and molecular characteristics generated during hydrolysis. The EPR results are presented in Figure 6 and are expressed as relative changes in radical signal intensity compared to the control.

**Figure 7:**
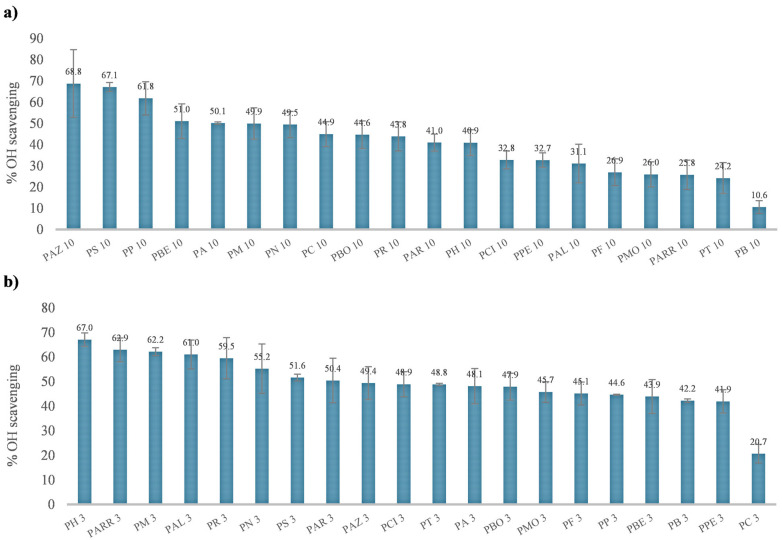
Resistance against oxidation by quantitative EPR, expressed as % inhibition of the ^●^OH radical by the Fenton reaction, for forty peptide fractions from common beans (see panels (**a**,**b**)).

Quantification of the scavenging response (Figure 7) revealed a broad distribution across samples, with ^●^OH inhibition values for the <3 kDa fractions ranging from 20.7 to 67.0% for PC and PH landraces, respectively. For the 3–10 kDa fractions, ^●^OH scavenging ranged from 10.6 to 68.8% for PB and PAZ landraces, respectively. For <3 kDa fractions, the PH landraces had the highest % ^●^OH scavenging (67.0%), followed by PARR (62.9%), PM (62.2%), and PAL (61%). For the 3–10 kDa fractions, the PAZ landraces had the highest % ^●^OH scavenging (68.8%), followed by PS (67.1%), PM (61.8%), and PBE (51.0%).

### 3.5. Statistical Analysis

The selection of statistical models was guided by assumption testing (Table S1), ensuring that each dataset was analyzed using methods consistent with its distributional properties. Normality was assessed using the Shapiro–Wilk and D’Agostino tests, and homogeneity of variances was evaluated using the Brown–Forsythe test (α = 0.05).

EPR data met both normality and homogeneity assumptions and were therefore analyzed using one-way ANOVA followed by Dunnett’s multiple comparisons test. In contrast, DPPH, FRAP, and ORAC datasets did not meet normality and/or variance homogeneity assumptions and were analyzed using the Kruskal–Wallis test followed by Dunn’s multiple comparisons test. Detailed multiple comparison results are provided in Table S2, while Table 2 summarizes statistical groupings using letter notation.

Pearson correlation analyses (Figure S1, Supplementary Materials) were performed to evaluate potential linear associations between soluble protein content and antioxidant activity measured by DPPH, FRAP, ORAC, and oxidation resistance assessed by EPR. No statistically significant linear relationships were detected for DPPH or EPR (r = 0.12 and r = 0.10, respectively; *p* > 0.05), indicating no evidence of association under the tested conditions.

In contrast, statistically significant but modest positive correlations were observed for FRAP and ORAC (r = 0.39 and r = 0.38, respectively; *p* < 0.05). However, the corresponding coefficients of determination (r^2^ = 0.14–0.15) indicate that soluble protein content accounts for only a limited proportion of the variability in antioxidant activity among landraces. These results suggest that antioxidant capacity cannot be explained solely by total soluble protein content or fraction abundance. Rather, additional compositional and structural factors, including peptide sequence, amino acid composition, and conformational characteristics, likely contribute substantially to the observed bioactivity differences.

## 4. Discussion

The variations in soluble protein content among landraces and fractions may help explain differences in peptide functionality and bioactivity. Unlike Dumas measurements performed on dry beans and protein isolates, which reflect total nitrogen, soluble protein is a better indicator of the functional availability of peptide fractions, since bioactivity depends on their presence in solution [42,45].

Fractions PPE, PA, PM, PAZ, PF, and PBE were the most prominent, particularly those below 3 kDa, with PPE being the most notable with 460 mg BSA/100 g of beans. These high values suggest a large proportion of small peptides with strong aqueous dispersibility, consistent with findings in legume hydrolysates where small peptides tend to be both highly soluble and reactive toward free radicals and metal ions [42,46]. In contrast, landraces such as PS, PN, and PARR showed lower solubility, lower than 60 mg BSA/100 g of beans, possibly due to more hydrophobic sequences or greater aggregation tendencies [47,48]. From a methodological standpoint, the quantification of soluble proteins provides a relevant indicator for peptide fractions, since theoretically soluble peptides can contribute proportionally to bioactivity in chemical and physiological systems. Therefore, these values will be used as a variable to correlate solubility with the antioxidant responses evaluated in this study.

The observed antioxidant activities across peptide fractions showed distinct patterns that are likely influenced by peptide composition, molecular size, and sequence-specific features. In the <3 kDa fraction, several landraces exhibited a strong radical-scavenging capacity, with PP showing the highest DPPH inhibition (39.6%), followed by PA (39.4%), PM (38.4%), and PBO (35.3%). In contrast, PS inhibition (12.9%), followed by PCI (13.1%), PAL (14.3%), and PARR (16.1%), at 360 μg/mL is consistent with the substantial landrace variability previously documented in common beans and reported for protein hydrolysates [49,50] and phenolic extracts from beans [16]. Also, 3–10 kDa fractions show that several landraces exhibited a strong radical-scavenging capacity, with PA showing the highest DPPH inhibition for PA (50.8%), followed by PP (48.9%), PPE (46.8%), and PM (42.5%). In contrast, PARR (13.1%), PCI (13.8%), PT (14.2%), and PAL (14.9%) exhibited significantly lower antioxidant activity. Our results agree with the literature, which describes potent antioxidant activity in intermediate-size legume peptides [51]. Zhang et al. [50] reported inhibition between 14 and 29% at 100 μg/mL for soy protein hydrolysate, while Chen et al. 2022 [49] reported 50% inhibition between 72 and 219 μg/mL as black bean protein hydrolysate doses.

FRAP results further highlighted the prominence of landraces. In the <3 kDa fraction, several landraces exhibited a higher reducing power, with PM showing the highest FRAP activity (1750 µmol TE/100 g beans), followed by PA (1420 µmol TE/100 g beans), PBO (1307 µmol TE/100 g beans), and PH (1097 µmol TE/100 g beans). In contrast, PCI (181 µmol TE/100 g beans), followed by PARR (211 µmol TE/100 g beans), PT (304 µmol TE/100 g beans), and PC (325.4 µmol TE/100 g beans) exhibited significantly lower antioxidant activity.

Similarly, in the 3–10 kDa fractions, several landraces exhibited a higher reducing power, with PA showing the highest FRAP activity (712 µmol TE/100 g beans), followed by PBO (528 µmol TE/100 g beans), PH (454 µmol TE/100 g beans), and PMO (405 µmol TE/100 g beans). In contrast, PARR (52 µmol TE/100 g beans), followed by PCI (57 µmol TE/100 g beans), PS (125 µmol TE/100 g beans), and PPE (162 µmol TE/100 g beans), exhibited significantly lower antioxidant activity, suggesting less favorable peptide compositions for metal-chelating and electron-donating mechanisms.

This behavior is consistent with previous reports indicating that peptide fractions within this molecular weight range can contribute to ferric ion reduction through electron donation and radical stabilization mechanisms, which are influenced by peptide size and amino acid composition [41]. In agreement with these observations, our results fall within the range of FRAP values reported for protein hydrolysates from common bean and other legume protein hydrolysates, including those described by Lopes et al. [52], who reported values between 44 and 870 μg/100 g, and by Sonklin et al. [53], who reported values between 61 and 242 μg/100 g protein.

ORAC analysis showed even greater differentiation among the local varieties. From the results presented in Table 2, among the peptide fractions <3 kDa, the PN landraces had the highest peroxyl radical scavenging capacity (5246 µmol TE/100 g beans), followed by PPE (2813 µmol TE/100 g beans), PA (2779 µmol TE/100 g beans), and PAR (2605 µmol TE/100 g beans). On the contrary, PP (868 µmol TE/100 g beans), PCI (1023 µmol TE/100 g beans), PAL (1042 µmol TE/100 g beans), and PARR (1105 µmol TE/100 g beans) showed lower ORAC activity in these fractions, suggesting a strong genetic influence on the antioxidant activity of peptides obtained from diverse bean varieties [16,54].

Also, the 3–10 kDa fractions exhibited higher ORAC values; the PA landraces had the highest peroxyl radical scavenging capacity (1838 µmol TE/100 g beans), followed by PBE (1775 µmol TE/100 g beans), PN (1663 µmol TE/100 g beans), and PB (1441 µmol TE/100 g beans). In contrast, PARR landraces (305 µmol TE/100 g beans), PAZ (418 µmol TE/100 g beans), PCI (421 µmol TE/100 g beans), and PP (529 µmol TE/100 g beans) exhibited lower ORAC values.

In agreement with the ORAC values obtained in this study (25.9–908 mmol TE/g protein DW), Mojica et al. [55] reported antioxidant capacities between 116 and 274 mmol TE/g protein DW in digested common bean proteins. Similarly, Oseguera-Toledo et al. [28] reported values ranging from 160 to 933 mmol TE/g protein DW in bean peptide fractions, depending on molecular weight and enzymatic treatment. These results support the influence of enzymatic hydrolysis and fractionation on the enrichment of bioactive peptide sequences with antioxidant potential [56,57].

When the two peptide fractions were compared directly, the <3 kDa peptides consistently exhibited higher antioxidant capacity than the 3–10 kDa peptides in ORAC and generally also in DPPH and FRAP assays, with only minor exceptions (e.g., PA in DPPH). This trend is consistent with extensive evidence indicating that low-molecular-weight peptides display superior antioxidant efficiency, which has been attributed to their greater molecular mobility, reduced steric hindrance, and increased exposure of reactive amino acid side chains, facilitating hydrogen donation and electron transfer mechanisms [58]. It should be noted that protein bands in the 15–25 kDa range were observed in SDS-PAGE; however, these higher molecular weight fractions were not further fractionated or functionally evaluated, as the present study focused specifically on ultrafiltration-derived peptide fractions (<3 kDa and 3–10 kDa). Future studies exploring the bioactivity of additional molecular weight ranges may provide further insight into the contribution of larger peptides or proteins.

Taken together, the ORAC, DPPH, and FRAP results demonstrate marked variability in antioxidant performance among landraces and peptide fractions, highlighting PA, PM, and PN as particularly promising sources of antioxidant peptides. Peptide extraction yields ranged from 3.73 to 10.39% for the <3 kDa fractions and from 1.33 to 4.74% for the 3–10 kDa fractions, indicating the potential for recovering bioactive peptides from selected landraces under laboratory conditions. These results emphasize the role of peptide composition and molecular characteristics in determining antioxidant efficiency, which is further examined through electron paramagnetic resonance (EPR) analysis under controlled radical-generating conditions.

Complementarily, the evaluation of RAO by EPR provided mechanistic insight into the antioxidant behavior of the peptide fractions beyond that obtained from conventional spectrophotometric assays. Unlike indirect methods such as DPPH, FRAP, or ORAC, EPR spin trapping enables the direct detection of short-lived radical species, allowing the assessment of hydroxyl radical scavenging under controlled conditions. In this context, the observed reduction in the DMPO–OH signal reflects the capacity of peptide fractions to directly interact with and neutralize highly reactive oxygen species.

The stronger attenuation of the EPR signal observed for the <3 kDa fraction indicates that low-molecular-weight peptides contribute substantially to hydroxyl radical scavenging under the present in vitro assay conditions. This behavior can be attributed to their higher diffusion rates, greater accessibility of reactive amino acid residues, and reduced steric hindrance, which facilitate interactions with radical species in the reaction system. Similar trends have been reported for bioactive peptides derived from legumes and other plant proteins, where smaller peptides frequently exhibit superior antioxidant performance [39,59].

The observed differences among landraces further highlight the influence of genetic variability on peptide composition and antioxidant potential. Variations in amino acid sequence, hydrophobicity, and the presence of residues capable of hydrogen donation or electron transfer likely account for the distinct EPR responses observed. These findings are consistent with previous reports indicating that peptide structure and sequence strongly modulate radical scavenging efficiency in food-derived hydrolysates [39,40].

Importantly, the EPR results complement the trends observed in the DPPH, FRAP, and ORAC assays, reinforcing the observation that peptide fractions enriched in <3 kDa peptides exhibit comparatively higher antioxidant performance across multiple mechanisms under the in vitro conditions evaluated. While spectrophotometric assays provide indirect measurements based on redox reactions, EPR confirms that these peptides are capable of directly quenching highly reactive oxygen species, supporting their potential functional relevance in oxidative stress mitigation.

In comparison with existing literature, the present EPR-based characterization represents a substantive and novel contribution. Although spin-trapping EPR has been widely applied to evaluate antioxidant dynamics in plant matrices [60], including herb extracts [59], Chilean hazelnut [61], and wines [62], its application to peptide systems remains relatively limited. Early peptide-focused work demonstrated •OH scavenging activity in dipeptides such as carnosine [63], but such studies have focused mainly on animal-derived sequences. More recent reviews describing antioxidant plant-based peptides and their mechanisms of action [64] emphasize that most evaluations still rely on colorimetric assays, with relatively few adopting direct radical detection.

To our knowledge, only limited evidence is available regarding the use of spin-trapping EPR for peptide fractions derived from common beans (*Phaseolus vulgaris* L.). Although antioxidant peptides from this species have been previously reported, including the study by Peng et al. [40], those analyses quantified radical scavenging only in relative terms. By combining ultrafiltration, peptide fractionation, and direct radical detection by EPR, the present work extends analytical approaches commonly applied in legume protein research. These results not only demonstrate the hydroxyl radical scavenging potential of common bean-derived peptides but also highlight the value of advanced techniques such as EPR for elucidating antioxidant mechanisms that cannot be fully explained by conventional chemical assays [65].

### 4.1. Limitations

This study has some limitations that should be considered when interpreting the results. All experiments were conducted under controlled laboratory conditions; therefore, the findings should not be directly extrapolated to industrial or commercial applications without further validation.

Soluble protein content is frequently used as a convenient parameter to estimate the presence of bioactive peptides. However, the results obtained in this study show that this assumption does not fully apply to antioxidant activity. The absence of clear associations with DPPH and EPR responses, and the presence of only weak correlations with FRAP and ORAC, indicate that protein solubility contributes only marginally to the overall antioxidant behavior of the peptide fractions. This suggests that antioxidant activity is driven mainly by peptide-specific features, including amino acid sequence and molecular weight distribution, and may also be influenced by co-extracted non-protein antioxidants.

From an applied perspective, soluble protein content remains a relevant parameter for technological and formulation purposes rather than as a predictor of bioactivity. High solubility favors dispersion and processing performance in food systems. However, it should be noted that these observations were obtained under controlled laboratory conditions, and further process optimization, scale-up validation, and techno-economic assessment would be required before considering potential industrial implementation.

Additionally, this study used commercial Chilean landraces from the same production year and region to minimize variability. Agronomic factors like soil or altitude may also influence peptide composition, and controlled studies with selected varieties are needed to evaluate these genotype × environment effects.

### 4.2. Future Perspectives

Given the analytical complexity and time-intensive nature of detailed peptide characterization, future work should prioritize a reduced set of landraces exhibiting the highest antioxidant activity. These selected samples will be further characterized by chromatographic separation coupled to high-resolution tandem mass spectrometry (LC-MS/MS using a QTOF mass analyzer), followed by bioinformatic analysis to determine peptide sequences and identify recurrent motifs associated with antioxidant activity, enabling a more precise understanding of structure–activity relationships. Additionally, it will be interesting to evaluate the gastrointestinal digestion stability of the peptide fractions, as this would support their potential as functional ingredients.

## 5. Conclusions

This work demonstrates that Chilean common bean (*Phaseolus vulgaris* L.) represents a promising source of antioxidant peptides under controlled laboratory conditions, with both molecular size and genotype influencing their bioactivity. In general, peptide fractions enriched in peptides below 3 kDa exhibited higher antioxidant activity in most in vitro assays, as well as higher extraction yields and solubility. These findings highlight the potential functional relevance of low-molecular-weight peptides and support their interest for further investigation, while additional studies will be necessary to assess their performance, stability, and feasibility in applied or scaled-up systems.

The significant differences observed among landraces further highlight the importance of genetic diversity as a driver of bean peptide bioactivity. Landraces such as PA, PM, PH, and PBO were identified as particularly promising sources of highly bioactive peptides of a low molecular weight. Additionally, some fractions within the 3–10 kDa range showed relevant antioxidant activity, suggesting that peptides of different sizes can contribute complementarily to the overall antioxidant potential of common bean protein hydrolysates.

By incorporating spin-trapping EPR into the experimental approach, this study provided direct evidence of hydroxyl radical scavenging by common bean peptide fractions. This technique strengthens the interpretation of antioxidant activity observed with conventional chemical assays and offers a valuable mechanistic perspective on how legume-derived peptides can exert their functional effects, expanding the analytical tools commonly used in this field.

Taken together, these findings suggest that peptide fractions enriched in low-molecular-weight peptides from common beans may represent promising plant-based ingredients with antioxidant potential under the experimental conditions evaluated. Future studies may be directed to identify the peptide sequences responsible for this activity and to perform structure–activity relationship studies of these potential functional ingredients based on common beans.

## Figures and Tables

**Table 1:** Total protein and yield protein extraction from twenty common bean landraces.

Landraces	Extraction Yield (%)	Protein in Seeds (g/100 g DW)	Protein in Isolates (g/100 g DW)	Protein Yield (%)
PS	17.86 ± 0.84	20.61 ± 0.17	72.19 ± 0.89	55.75 ± 0.53
PH	18.24 ± 0.21	18.16 ± 0.06	70.8 ± 3.9	62.79 ± 0.15
PN	21.76 ± 0.10	20.64 ± 0.20	71.3 ± 2.7	66.20 ± 0.80
PR	18.97 ± 0.22	20.24 ± 0.21	71.6 ± 2.8	57.51 ± 0.73
PF	25.17 ± 0.24	20.25 ± 0.06	69.5 ± 3.4	75.81 ± 0.23
PC	17.11 ± 0.12	18.99 ± 0.16	74.35 ± 0.44	58.79 ± 0.38
PA	19.84 ± 0.43	19.85 ± 0.24	74.6 ± 2.1	65.39 ± 0.89
PBE	21.05 ± 0.13	20.45 ± 0.16	76.5 ± 1.8	69.50 ± 0.17
PP	25.62 ± 0.79	20.89 ± 0.33	59.8 ± 4.6	67.29 ± 0.17
PT	15.32 ± 0.28	16.92 ± 0.08	74.5 ± 2.9	59.51 ± 0.01
PB	17.04 ± 0.79	17.24 ± 0.25	74.6 ± 1.6	66.45 ± 0.63
PAR	16.02 ± 0.10	15.18 ± 0.37	65.8 ± 1.2	60.16 ± 0.86
PM	20.21 ± 0.30	19.25 ± 0.34	73.3 ± 5.0	68.63 ± 0.20
PAL	23.06 ± 0.09	20.77 ± 0.39	71.63 ± 0.91	71.36 ± 0.95
PBO	25.14 ± 0.18	19.36 ± 0.29	66.8 ± 4.0	76.1 ± 1.0
PMO	22.84 ± 0.17	18.34 ± 0.36	71.0 ± 3.6	77.9 ± 1.3
PAZ	18.90 ± 0.13	19.94 ± 0.23	80.79 ± 0.53	67.26 ± 0.56
PPE	19.32 ± 0.27	20.16 ± 0.27	77.4 ± 3.0	64.70 ± 0.90
PCI	26.40 ± 0.59	22.75 ± 0.19	74.0 ± 2.4	75.17 ± 0.23
PARR	21.82 ± 0.16	21.78 ± 0.56	81.3 ± 2.0	72.99 ± 0.35

Extraction yield (%) = grams of protein isolate obtained per 100 g of initial dry seeds; protein yield (%) = grams of protein recovered in the isolate relative to the total protein content present in 100 g of initial dry seeds; protein in seeds (g/100 g DW) = grams of protein determined in 100 g of whole dry seeds; protein in isolates (g/100 g DW) = grams of protein determined in 100 g of isolated protein fraction.

**Table 2:** Antioxidant activities by DPPH, FRAP, and ORAC assays of forty peptides from common beans.

Landraces	Fraction (kDa)	Peptide Extraction Yield (%)	DPPH (% at 380 µg/mL)	FRAP (µmol TE/100 g Beans)	ORAC (µmol TE/100 g Beans)
PS	10-3	1.33	20.0 ± 2.9 ^a^	125 ± 14 ^a^	645 ± 27 ^a^
	≤3	3.75	12.87 ± 0.79 ^a^	425 ± 36 ^a^	2164 ± 115 ^a^
PH	10-3	3.50	20.5 ± 1.8 ^a^	454 ± 36 ^a^	983 ± 98 ^a^
	≤3	8.01	25.1 ± 1.0 ^a^	1097 ± 82 ^a^	1893 ± 220 ^a^
PN	10-3	2.45	27.8 ± 1.5 ^a^	296 ± 16 ^a^	1663 ± 172 ^a^
	≤3	7.48	30.1 ± 3.5 ^a^	723 ± 42 ^a^	5246 ± 576 ^b^
PR	10-3	1.79	37.9 ± 1.1 ^a^	224 ± 16 ^a^	1231 ± 11 ^a^
	≤3	4.38	34.1 ± 3.1 ^a^	524 ± 41 ^a^	1992 ± 267 ^a^
PF	10-3	3.60	33.9 ± 1.2 ^a^	346 ± 36 ^a^	1088 ± 17 ^a^
	≤3	7.76	32.7 ± 1.2 ^a^	1073 ± 21 ^a^	1809 ± 127 ^a^
PC	10-3	2.26	22.85 ± 0.40 ^a^	171 ± 21 ^a^	638 ± 15 ^a^
	≤3	3.73	31.3 ± 4.6 ^a^	325 ± 22 ^a^	1666 ± 64 ^a^
PA	10-3	4.74	50.8 ± 5.1 ^b^	712 ± 30 ^a^	1838 ± 40 ^a^
	≤3	10.39	39.4 ± 3.0 ^a^	1420.0 ± 4.5 ^a^	2779 ± 96 ^b^
PBE	10-3	4.35	14.83 ± 0.40 ^a^	376 ± 24 ^a^	1775 ± 26 ^a^
	≤3	6.34	19.9 ± 3.0 ^b^	682 ± 52 ^a^	1412 ± 89 ^a^
PP	10-3	1.74	48.9 ± 7.6 ^a^	296 ± 24 ^a^	529 ± 9.2 ^a^
	≤3	3.74	39.6 ± 2.9 ^a^	828 ± 34 ^a^	868 ± 42 ^a^
PT	10-3	2.61	14.24 ± 0.22 ^a^	252 ± 11 ^a^	1068 ± 48 ^a^
	≤3	4.10	17.66 ± 0.39 ^a^	303.6 ± 8.9 ^a^	1652 ± 49 ^a^
PB	10-3	3.23	22.8 ± 1.2 ^a^	295 ± 15 ^a^	1441 ± 195 ^a^
	≤3	5.32	17.8 ± 1.1 ^a^	500 ± 27 ^a^	2249 ± 230 ^a^
PAR	10-3	2.19	25.7 ± 1.5 ^a^	242 ± 17 ^a^	899 ± 14 ^a^
	≤3	7.77	21.4 ± 1.1 ^a^	756 ± 38 ^a^	2605 ± 72 ^b^
PM	10-3	2.07	42.5 ± 1.7 ^a^	339 ± 15 ^a^	660 ± 19 ^a^
	≤3	8.93	38.37 ± 0.88 ^a^	1750 ± 52	2173.4 ± 4.5 ^b^
PAL	10-3	2.69	14.86 ± 0.39 ^a^	277 ± 12 ^a^	686 ± 32 ^a^
	≤3	5.00	14.30 ± 0.11 ^a^	537 ± 21 ^a^	1042 ± 50 ^a^
PBO	10-3	3.11	40.1 ± 1.0 ^a^	528 ± 24 ^a^	930.0 ± 7.0 ^a^
	≤3	7.73	35.3 ± 1.6 ^a^	1307 ± 59 ^a^	1726 ± 75 ^a^
PMO	10-3	2.91	38.2 ± 2.8 ^a^	405 ± 16 ^a^	731 ± 16 ^a^
	≤3	6.60	28.4 ± 2.1 ^a^	901 ± 50 ^a^	1323 ± 49 ^a^
PAZ	10-3	2.00	20.02 ± 1.40 ^a^	190.5 ± 7.5 ^a^	418 ± 26 ^a^
	≤3	6.33	16.70 ± 0.92 ^b^	636 ± 31 ^a^	1771 ± 22 ^a^
PPE	10-3	2.24	46.77 ± 0.75 ^a^	162 ± 16 ^a^	572 ± 64 ^a^
	≤3	9.86	34.58 ± 0.11 ^a^	544 ± 28 ^a^	2813 ± 212 ^b^
PCI	10-3	1.56	13.81 ± 0.97 ^a^	56.6 ± 2.6 ^a^	421 ± 51 ^a^
	≤3	4.70	13.06 ± 0.81 ^a^	180.7 ± 7.4 ^a^	1023 ± 17 ^a^
PARR	10-3	1.44	13.06 ± 0.99 ^a^	52.2 ± 3.1 ^a^	305 ± 10 ^a^
	≤3	5.74	16.1 ± 1.4 ^a^	211.2 ± 9.8 ^a^	1105 ± 99 ^a^

Peptide extraction yield (%) = dry mass of peptides recovered relative to the initial mass of the bean substrate, expressed as a percentage and normalized to the protein content of the substrate. Different superscript letters within the same column indicate significant differences among landraces according to the appropriate post hoc test for each assay (Dunnett for FRAP and EPR; Dunnett T3 for ORAC to account for unequal variances). Means that share the same letter are not significantly different from the control at *p* < 0.05. See Table S1 for statistical models and Table S2 for complete pairwise comparisons.

## Data Availability

The original contributions presented in the study are included in the article and the Supplementary Material. Further inquiries can be directed to the corresponding author.

## Supplementary Materials

The following supporting information can be downloaded at: https://www.mdpi.com/article/10.3390/antiox15030376/s1. Table S1. Statistical assumption testing and model selection for antioxidant activity assays. Normality was evaluated using Shapiro–Wilk and D’Agostino tests. Homogeneity of variances was assessed using the Brown–Forsythe test. A significance level of α = 0.05 was applied for all assumption tests. When both assumptions were met, one-way ANOVA followed by Dunnett’s post hoc test was used. When variance homogeneity was violated, Welch’s ANOVA followed by Dunnett’s T3 post hoc test was applied. Analyses were performed using GraphPad Prism v.8.0.1. Table S2. Statistical differences between sample means for DPPH, FRAP, ORAC, and EPR assays were evaluated using one-way ANOVA or Welch’s ANOVA as appropriate (see Table S1), followed by Dunnett-type multiple comparison tests. For each assay, the control group was defined as the sample exhibiting the highest antioxidant value: PA 10 for DPPH, PM 3 for FRAP, PN 3 for ORAC, and PAZ 10 for EPR. Significance levels were defined as *p* < 0.05 (*), *p* < 0.01 (**), *p* < 0.001 (***), and *p* < 0.0001 (****); Figure S1. Pearson correlation statistical analysis between soluble protein content and antioxidant activity using the DPPH, FRAP, ORAC, and EPR methods.

## Abbreviations

The following abbreviations are used in this manuscript:

EPR	Electron Paramagnetic Resonance
ORAC	Oxygen Radical Absorbance Capacity
DPPH	2,2-Diphenyl-1-picrylhydrazyl
FRAP	Ferric Reducing Antioxidant Power
ROS	Reactive Oxygen Species
NCDs	Non-communicable Diseases
BSA	Bovine Serum Albumin
DMPO	5,5-Dimethyl-1-pyrroline N-oxide
