# Supplementary material for: Antioxidant Activity and Resistance Against Oxidation of Peptide Fractions from Common Bean (Phaseolus vulgaris L.) Landraces Assessed by EPR and Chemical Assays
Source: Antioxidants (Basel). 2026 Mar 18;15(3):376. doi: 10.3390/antiox15030376 (PMC13023929; doi:10.3390/antiox15030376)
Supplement: Supplementary file 1 [file antioxidants-15-00376-s001.zip › antioxidants-4141991-supplementary.pdf]

Supplementary Materials

Antioxidant Activity and Resistance Against Oxidation of Peptide Fractions from Common Bean (*Phaseolus vulgaris* L.) Landraces Assessed by EPR and Chemical Assays

Katherine Márquez-Calvo [1,*], Guillermo Schmeda-Hirschmann [2], Felipe Leyton [2], Felipe Ávila [3], Pablo Salgado [4], Victoria Melin [5], David Contreras [5] and Gipsy Tabilo-Munizaga [1]

1 Department of Food Engineering, Faculty of Health and Food Science, University of Bío-Bío, Av. Andrés Bello 720, Chillán 4051381, Chile; gtabilo@ubiobio.cl
2 Laboratorio de Química de Productos Naturales, Instituto de Química de Recursos Naturales, Universidad de Talca, Campus Lircay, Talca 3480094, Chile; schmeda@utalca.cl (G.S.-H.); frleyton@uc.cl (F.L.)
3 Department of Nutrition and Food Science, School of Nutrition and Dietetics, Health Science Faculty, Campus Lircay, University of Talca, Talca 3480094, Chile; favilac@utalca.cl
4 Departamento de Ingeniería Civil, Facultad de Ingeniería, Universidad Católica de la Santísima Concepción, Concepción 4090541, Chile; psalgado@ucsc.cl
5 Departamento de Química Analítica e Inorgánica, Facultad de Ciencias Químicas, Universidad de Concepción, Campus Universitario, Concepción 4070409, Chile; victoriamelin@udec.cl (V.M.); dcontrer@udec.cl (D.C.)
* Correspondence: kmarquez@ubiobio.cl; Tel.: +56-422-463137

Table S1. Statistical assumption testing and model selection for antioxidant activity assays. Normality was evaluated using Shapiro–Wilk and D'Agostino tests. Homogeneity of variances was assessed using the Brown–Forsythe test. A significance level of α = 0.05 was applied for all assumption tests. When both assumptions were met, one-way ANOVA followed by Dunnett's post hoc test was used. When variance homogeneity was violated, Welch's ANOVA followed by Dunnett's T3 post hoc test was applied. Analyses were performed using GraphPad Prism v.8.0.1.

Assay	Normality tests (p-values; assumption met?)	Brown–Forsythe test p-value	Variance homogeneity	Statistical model	Post hoc test
**DPPH**	Shapiro–Wilk: p= 0.016; No<br>D'Agostino: p= 0.072; Yes	p= 0.514	Homogeneous	One-way ANOVA	Dunnett
**FRAP**	Shapiro–Wilk: p< 0.0002, No<br>D'Agostino: p< 0.0004, No	p= 0.992	Homogeneous	One-way ANOVA	Dunnett
**EPR**	Shapiro–Wilk: p= 0.159; Yes<br>D'Agostino: p= 0.422; Yes	p= 0.961	Homogeneous	One-way ANOVA	Dunnett

**ORAC**	Shapiro–Wilk: p< 0.0001; No<br>D'Agostino: p< 0.0001; No	p= 0.0012	Heterogeneous	Welch ANOVA	Dunnett T3

For normality assessment, "Yes" indicates that the assumption was met ($p > 0.05$), whereas "No" indicates violation. Brown–Forsythe p-values correspond to variance homogeneity testing. Post hoc comparisons were performed against the selected reference sample.

Table S2. Statistical differences between sample means for DPPH, FRAP, ORAC, and EPR assays were evaluated using one-way ANOVA or Welch's ANOVA as appropriate (see Table S1), followed by Dunnett-type multiple comparison tests. For each assay, the control group was defined as the sample exhibiting the highest antioxidant value: PA 10 for DPPH, PM 3 for FRAP, PN 3 for ORAC, and PAZ 10 for EPR.

**Compared sample**	**p-value DPPH**		**p-value FRAP**		**p-value ORAC**		**p-value EPR**	
PS 10	<0.0001	****	<0.0001	****	*	0.0241	ns	0.9996
PS 3	<0.0001	****	<0.0001	****	*	0.0465	*	0.0272
PH 10	<0.0001	****	<0.0001	****	*	0.0247	****	<0.0001
PH 3	<0.0001	****	<0.0001	****	*	0.0274	ns	0.9996
PN 10	<0.0001	****	<0.0001	****	*	0.0282	**	0.0079
PN 3	<0.0001	****	<0.0001	****	-	-	ns	0.1600
PR 10	<0.0001	****	<0.0001	****	*	0.0318	***	0.0002
PR 3	<0.0001	****	<0.0001	****	*	0.0246	ns	0.6775
PF 10	<0.0001	****	<0.0001	****	*	0.0297	****	<0.0001
PF 3	<0.0001	****	<0.0001	****	*	0.0359	***	0.0004
PC 10	<0.0001	****	<0.0001	****	*	0.0242	***	0.0004
PC 3	<0.0001	****	<0.0001	****	*	0.0379	****	<0.0001
PA 10	-	-	<0.0001	****	*	0.0431	*	0.0115

PA 3	<0.0001	****	<0.0001	****	ns	0.0759	**	0.0033
PBE 10	<0.0001	****	<0.0001	****	*	0.0420	*	0.0194
PBE 3	0.9984	ns	<0.0001	****	*	0.0315	***	0.0002
PP 10	<0.0001	****	<0.0001	****	*	0.0232	ns	0.9535
PP 3	<0.0001	****	<0.0001	****	*	0.0262	***	0.0003
PT 10	<0.0001	****	<0.0001	****	*	0.0286	****	<0.0001
PT 3	<0.0001	****	<0.0001	****	*	0.0385	**	0.0051
PB 10	<0.0001	****	<0.0001	****	*	0.0223	****	<0.0001
PB 3	<0.0001	****	<0.0001	****	*	0.0349	****	<0.0001
PAR 10	<0.0001	****	<0.0001	****	*	0.0272	****	<0.0001
PAR 3	0.0008	***	<0.0001	****	ns	0.0686	*	0.0137
PM 10	<0.0001	****	<0.0001	****	*	0.0244	*	0.0101
PM 3	<0.0001	****	-	-	ns	0.0537	ns	0.9737
PAL 10	<0.0001	****	<0.0001	****	*	0.0244	****	<0.0001
PAL 3	<0.0001	****	<0.0001	****	*	0.0281	ns	0.8846
PBO 10	<0.0001	****	<0.0001	****	*	0.0277	***	0.0003
PBO 3	<0.0001	****	<0.0001	****	*	0.0385	**	0.0029
PMO 10	<0.0001	****	<0.0001	****	*	0.0252	****	<0.0001
PMO 3	<0.0001	****	<0.0001	****	*	0.0323	***	0.0006
PAZ 10	<0.0001	****	<0.0001	****	*	0.0220	-	-
PAZ 3	0.4292	ns	<0.0001	****	*	0.0420	**	0.0075
PPE 10	<0.0001	****	<0.0001	****	*	0.0222	****	<0.0001
PPE 3	<0.0001	****	<0.0001	****	ns	0.0610	****	<0.0001
PCI 10	<0.0001	****	<0.0001	****	*	0.0213	****	<0.0001
PCI 3	<0.0001	****	<0.0001	****	*	0.0288	**	0.0055
PARR 10	<0.0001	****	<0.0001	****	*	0.0212	****	<0.0001
PARR 3	<0.0001	****	<0.0001	****	*	0.0262	ns	0.9922

Significance levels were defined as $p< 0.05$ (*). $p< 0.01$ (**). $p< 0.001$ (***). $p < 0.0001$ (****); ns= non-significant.

Figure S1. Pearson correlation statistical analysis between soluble protein content and antioxidant activity using the DPPH. FRAP. ORAC. and EPR methods.

a)

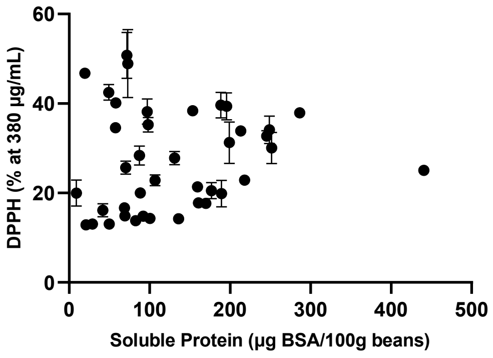

Pearson r: 0.119 (ns)

b)

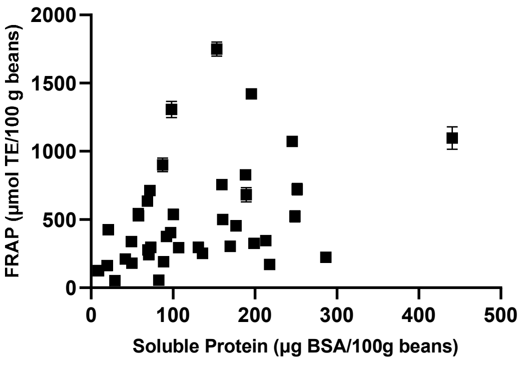

Pearson r: 0.387 (*)

c)

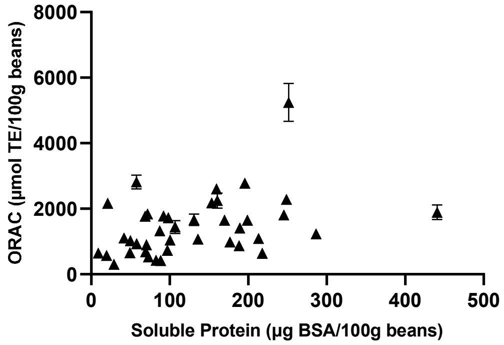

Pearson r: 0.379 (*)

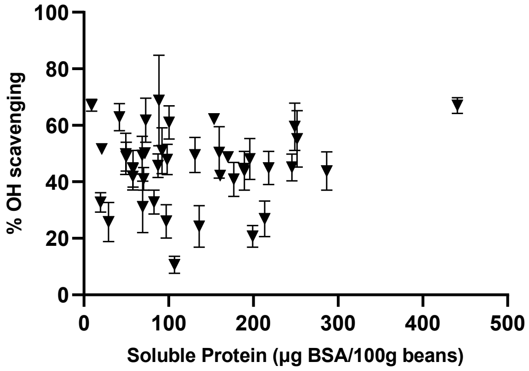

R-squared: 0.0959 (ns)

ns: non-significant; *: significant.
